# High-Pressure Water Injection Injury to the Hand: An Underestimated Mechanism of Compartment Syndrome

**DOI:** 10.7759/cureus.90383

**Published:** 2025-08-18

**Authors:** Abdul Rehman, Muhammad Yahya Khan, Raja Ansar Hameed, Fazeelah Bibi, Muhammad Amjad

**Affiliations:** 1 Department of Emergency Medicine, St. Luke's General Hospital, Killkenny, IRL; 2 Department of General Surgery, Allied Hospital, Faisalabad, PAK; 3 Department of Pediatric Surgery, Children Hospital, Faisalabad, PAK; 4 Department of Emergency Medicine, St. Luke's General Hospital, Kilkenny, IRL; 5 Department of Emergency Medicine, Pakistan Institute of Medical Sciences Hospital, Islamabad, PAK

**Keywords:** digital compartment syndrome, fasciotomy, hand trauma, high-pressure injection injury, power washer injury, subcutaneous emphysema

## Abstract

High-pressure injection injuries, although rare, pose a significant risk of severe soft tissue damage and compartment syndrome, especially when involving the hand and fingers. We present the case of a 25-year-old male patient who accidentally sustained a high-pressure water jet injury while using a power washer, leading to digital compartment syndrome of the thumb and index finger. Prompt recognition, early surgical decompression, and multidisciplinary care led to a favorable outcome. This case highlights the deceptive appearance of such injuries and underscores the need for urgent surgical intervention to prevent permanent functional impairment or limb loss. Early diagnosis, supported by clinical findings and radiological imaging, is crucial to improving outcomes in high-pressure injection injuries.

## Introduction

High-pressure injection injuries to the hand are uncommon but represent a surgical emergency due to the high risk of compartment syndrome and irreversible tissue damage. These injuries can result from paint guns, grease injectors, or water jets and are characterized by small entry wounds that belie the extent of internal damage [1,2]. Water-based injection injuries are less commonly reported than grease or paint but can be equally destructive due to mechanical and chemical effects [3]. The critical nature of early recognition and prompt decompressive fasciotomy cannot be overemphasized, as delays may lead to poor perfusion, necrosis, infection, or amputation [4].

## Case presentation

A 25-year-old, right-hand-dominant, male patient with no significant past medical or surgical history presented to the emergency department two hours after sustaining an accidental injury to his right hand. He was cleaning a drainage pipe at a farmhouse using a high-pressure water power washer when the injury occurred. He reported immediate pain, swelling, and numbness in his right thumb and index finger.

**Figure 1:**
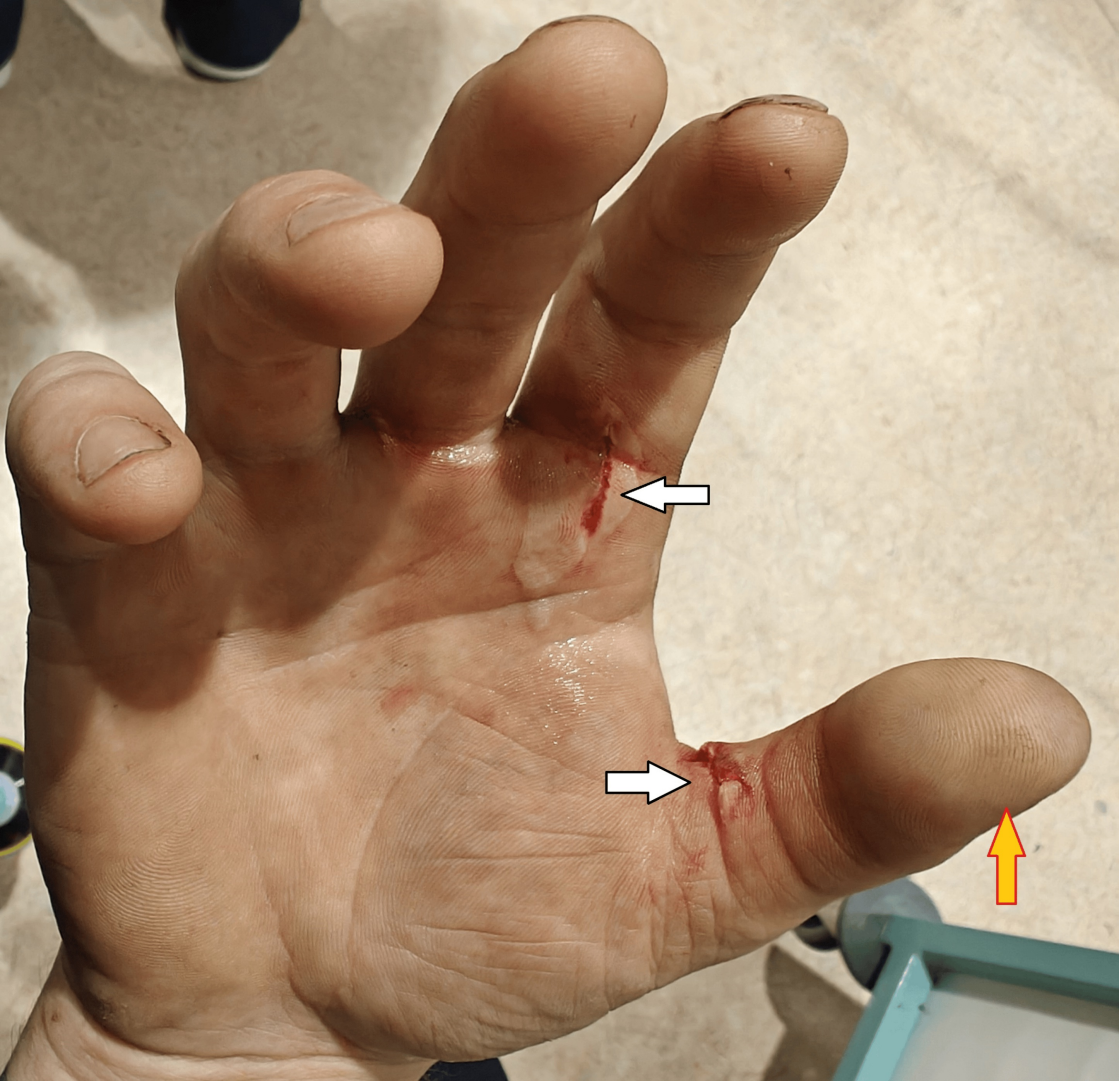
Superficial lacerations at the base of index finger and thumb (white arrows); pale discoloration of the thumb tip is noted (yellow arrow)

**Figure 2:**
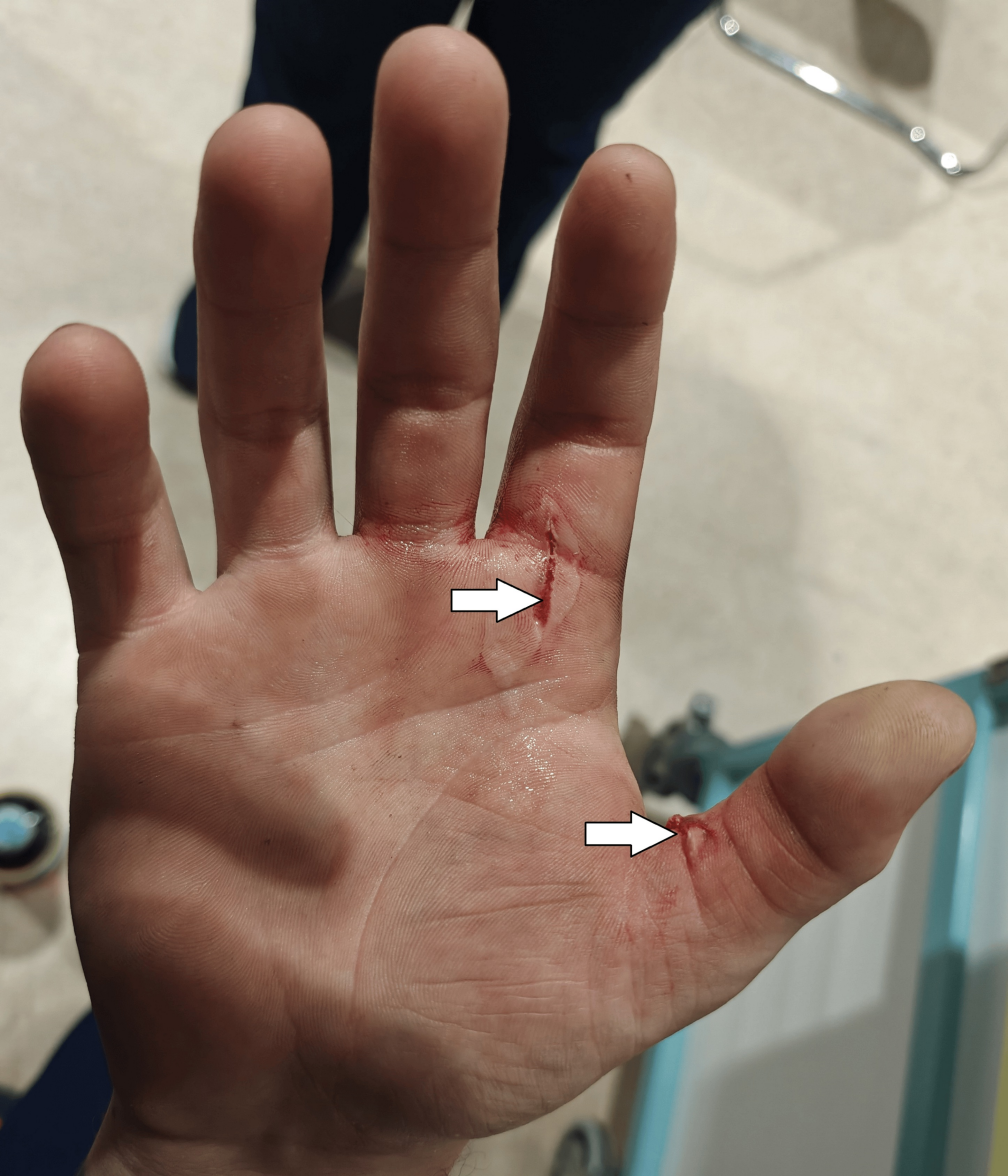
Superficial lacerations at the base of the right index finger and thumb (white arrows)

**Figure 3:**
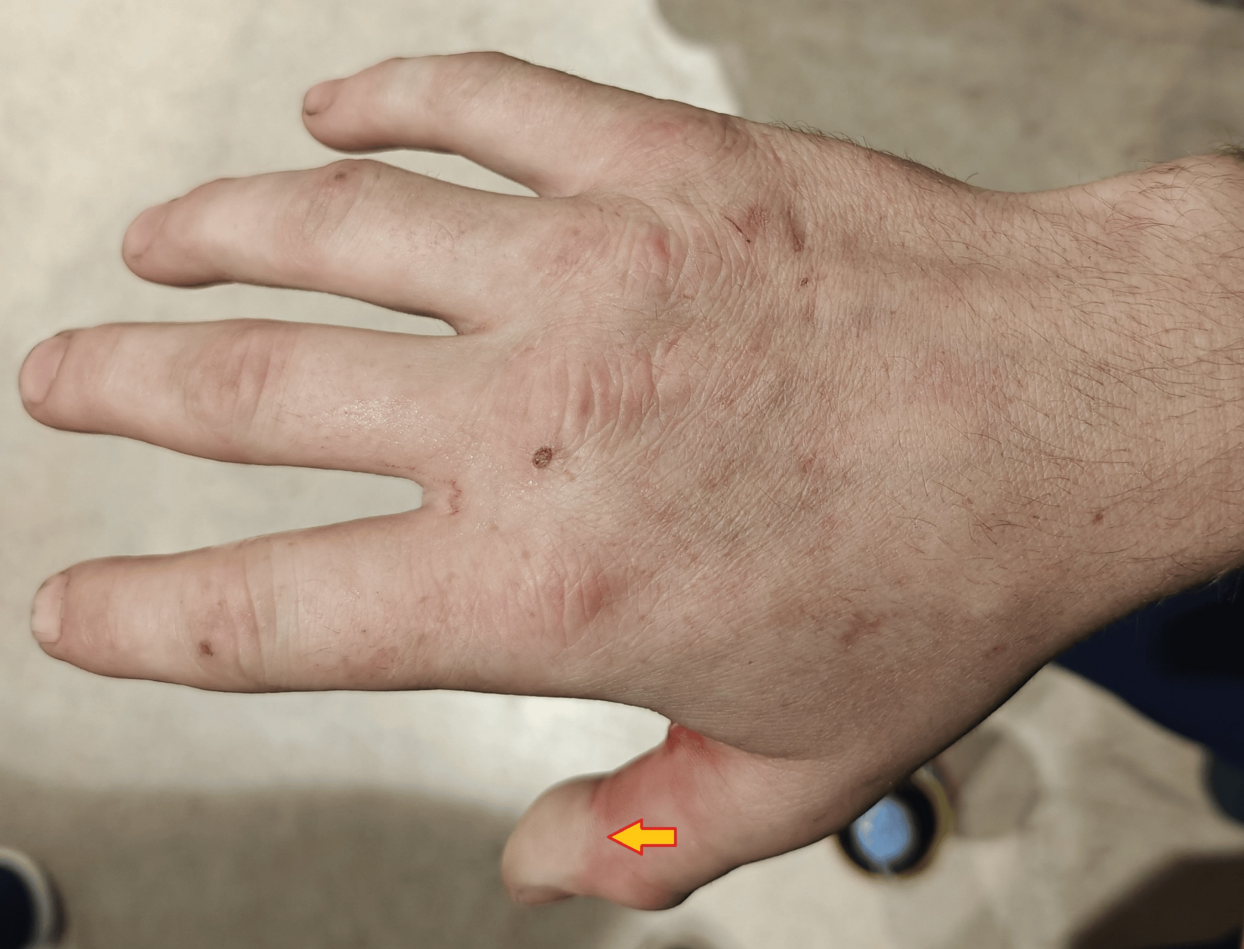
Pale discoloration of the right distal thumb (yellow arrow)

On examination, he had swelling and tense tenderness over the right thumb and index finger with two superficial lacerations on the palmar aspects of the proximal phalanges (Figures 1, 2). Crepitus was palpable in the thumb, index finger, and first web space. Active finger flexion and extension were limited due to pain and swelling, although he could initiate movement at the metacarpophalangeal joint (MCPJ), proximal interphalangeal joint (PIPJ), and distal interphalangeal joint (DIPJ) in the index finger, and MCPJ and interphalangeal (IPJ) in the thumb. Capillary refill was delayed at the thumb tip, which appeared pale and cooler than the adjacent digits (Figure 3). Sensory examination revealed decreased light touch and pinprick sensation over the tip of the thumb.

**Figure 4:**
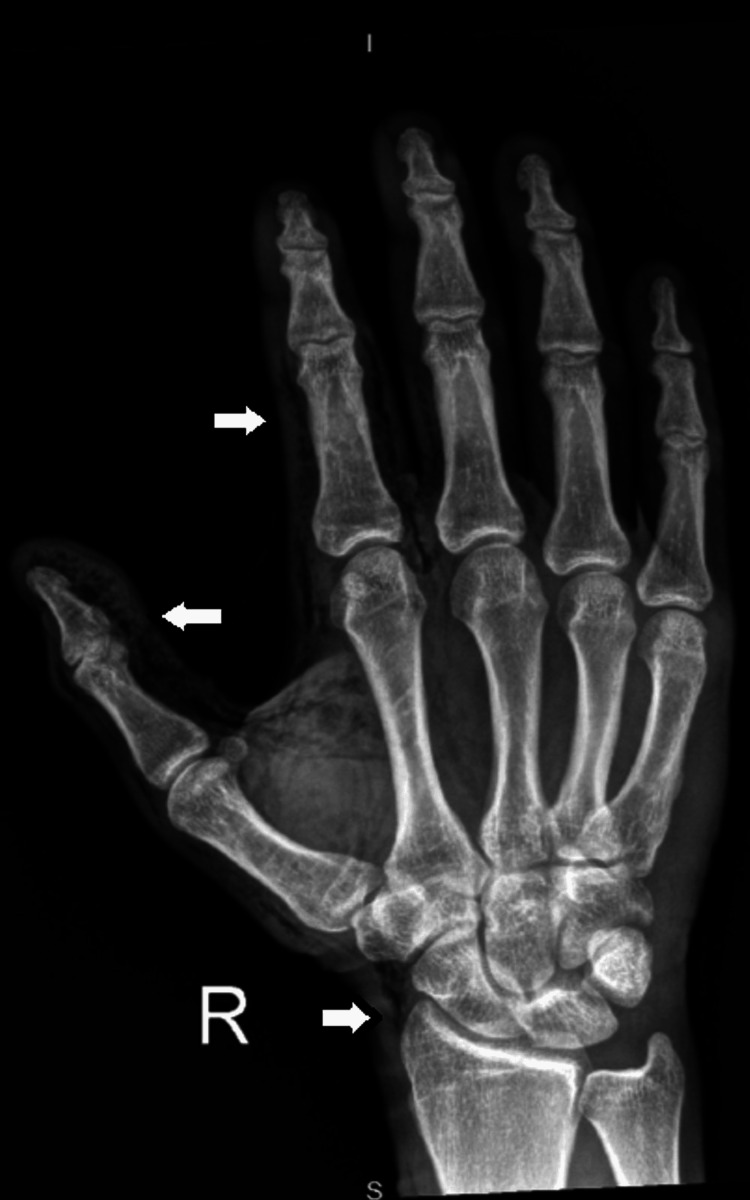
An X-ray showing extensive subcutaneous edema and emphysema (white arrows) within the right thumb, index finger, and middle finger, which extends to the wrist and palmar soft tissue.

**Figure 5:**
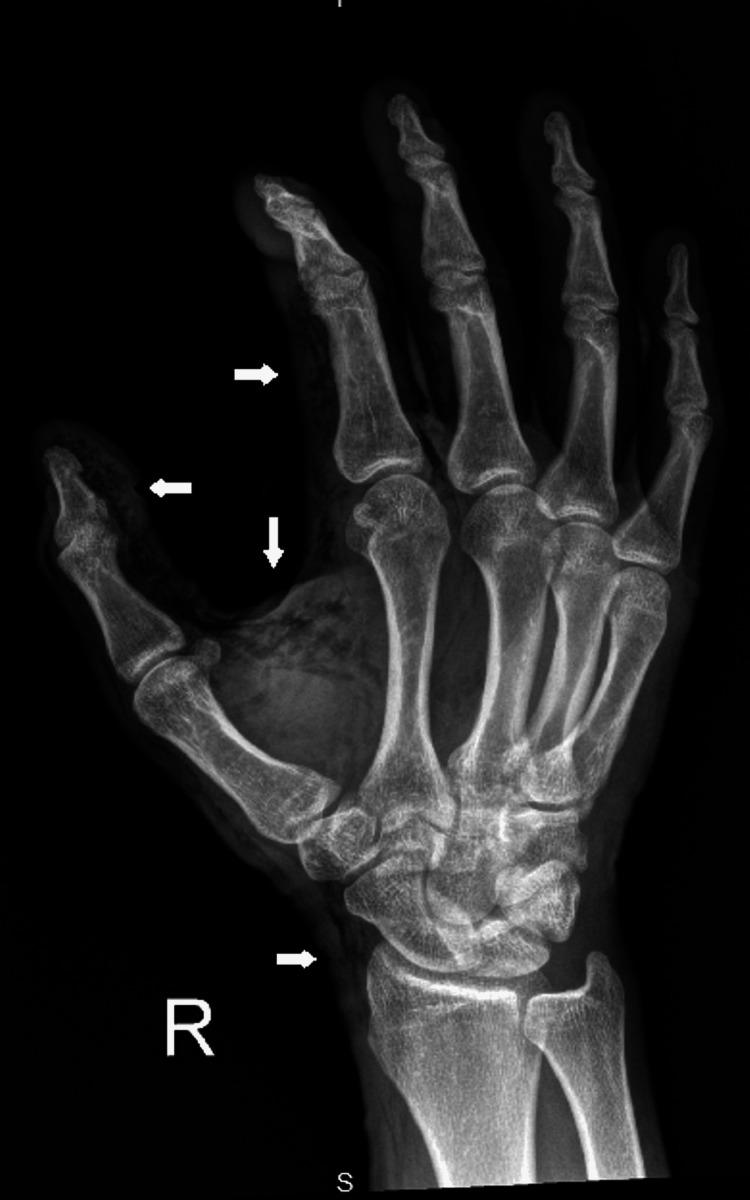
An X-ray showing extensive subcutaneous edema and emphysema (white arrows) within the right thumb, index finger, and middle finger, which extends to the wrist and palmar soft tissue.

An intravenous line was established, and the patient was administered analgesia, a tetanus toxoid booster, and intravenous antibiotics. Radiographs of the right hand revealed extensive subcutaneous emphysema in the thumb and index finger and extending into the palmar soft tissues and wrist (Figures 4, 5), confirming the suspicion of a high-pressure injection injury with compartment syndrome.

The plastic and hand surgery team was consulted urgently. The patient underwent surgical fasciotomy and digital compartment decompression with bilateral incisions over the midlines of the involved phalanges of the thumb and index finger. Postoperatively, there was immediate improvement in pain and sensation, and capillary refill improved. Although the thumb tip remained pale on the first day, it regained normal color and warmth by postoperative day two.

The patient was discharged on postoperative day two with instructions for wound care and signs of infection. The wounds were left to heal by secondary intention. At follow-up, the patient demonstrated complete recovery without functional loss.

## Discussion

Injuries caused by high-pressure injection are infrequent but can result in significant morbidity, especially when they involve the hand. These injuries often appear deceptively minor on initial presentation due to small or superficial entry wounds, yet they can mask extensive internal damage. The forces involved in high-pressure injuries, whether from grease, paint, or water, can drive substances deep into soft tissue compartments, leading to tissue disruption, vascular compromise, and compartment syndrome [1,2].

In this case, the injury was caused by high-pressure water, a medium generally perceived as non-toxic. Nonetheless, water under extreme pressure can induce considerable soft tissue trauma through mechanical dissection alone. This results in swelling, vascular compression, and the introduction of air and microbes into normally sterile tissue planes [3]. Though less chemically harmful than substances like paint or hydraulic oil, water can still trigger severe inflammation, ischemia, and infection due to the force of injection.

Early signs of compartment syndrome, such as pain disproportionate to the injury, swelling, sensory changes, and decreased capillary refill, were present in our patient. These clinical features, especially when combined with imaging showing subcutaneous air, should prompt urgent surgical evaluation [4]. Although not all “classic” symptoms of compartment syndrome may be evident early on, increasing pain and neurological changes are especially concerning and require swift intervention [5].

Plain radiographs are essential in such scenarios. They can detect subcutaneous emphysema, help identify retained foreign material, and rule out fractures. In our case, radiographs confirmed widespread air tracking through the thumb, index finger, and palm, consistent with high-pressure fluid entry [6].

Definitive treatment involves timely surgical decompression of affected compartments. Digital fasciotomy, as performed in our case, helps relieve elevated pressures and restore blood flow. Delayed intervention significantly increases the risk of permanent damage, including tissue necrosis and possible digit loss [7,8]. In high-pressure injuries, early fasciotomy has been shown to improve functional outcomes and reduce the need for amputation.

Postoperative management focuses on infection prevention, pain control, and close vascular monitoring. Given the contamination risk, wounds are commonly left open and managed with regular dressings to allow healing by secondary intention [9]. Antibiotics and tetanus prophylaxis are standard, especially in cases involving water or organic material, as was true for our patient who was working on a farm.

Importantly, there is a need for greater public and occupational awareness of the hazards posed by high-pressure equipment. The lack of protective gear and casual handling of such devices in non-industrial settings, like farms or home maintenance, contributes to under-recognized but serious injuries. Educational efforts on proper equipment use and the dangers of high-pressure tools may help reduce incidence and improve outcomes through early recognition and treatment [10].

## Conclusions

This case demonstrates that high-pressure water injection injuries to the hand can rapidly progress to digital compartment syndrome, even when external wounds appear minor. Prompt clinical recognition, supported by radiographic evaluation, is essential for initiating life- and limb-saving surgical intervention. Early fasciotomy can reverse ischemic changes and prevent long-term disability or amputation. Clinicians should maintain a high degree of suspicion in similar cases, especially when patients present with pain out of proportion, altered sensation, and delayed capillary refill. In addition to medical management, public awareness of the risks associated with power washers and similar equipment is critical in preventing such injuries. Interdisciplinary collaboration between emergency physicians, radiologists, and hand surgeons is key to achieving favorable outcomes.
